# Zebrafish Model for Studying Dexamethasone-Induced Muscle Atrophy and Preventive Effect of Maca (*Lepidium meyenii*)

**DOI:** 10.3390/cells10112879

**Published:** 2021-10-25

**Authors:** Bomi Ryu, Jun-Geon Je, You-Jin Jeon, Hye-Won Yang

**Affiliations:** 1 Department of Marine Life Science, Jeju National University, Jeju 63243, Korea; bmryu@jejunu.ac.kr (B.R.); wpwnsrjs@naver.com (J.-G.J.); 2 Healthy Seafood Research Center, Jeju National University, Jeju 63243, Korea; 3 Marine Science Institute, Jeju National University, Jeju 63333, Korea

**Keywords:** zebrafish, muscle atrophy, dexamethasone, maca

## Abstract

Loss of myofibers during muscle atrophy affects functional capacity and quality of life. Dexamethasone, an inducer of rapid atrophy of skeletal myofibers, has been studied as a glucocorticoid receptor in muscle atrophy or motor neurodegeneration. In this study, we examined dexamethasone-induced muscle atrophy using zebrafish (*Danio rerio*), a vertebrate model, and assessed whether administration of *Lepidium meyenii* (maca) as a dietary supplement can prevent muscle atrophy. Changes in skeletal myofibers in zebrafish were evaluated after exposure to dexamethasone for different periods and at different concentrations. Under optimized conditions, zebrafish pre-fed with maca for 3 days were exposed to 0.01% dexamethasone for 1 h/day for 7 days. Thereafter, myofiber loss, damaged muscle contractile proteins, and abnormal exploratory behavior due to the structural and functional impairment of skeletal muscle associated with muscle atrophy were investigated using hematoxylin–eosin, immunofluorescence staining, and behavioral analyses. Our findings suggest that dexamethasone induces muscle atrophy in zebrafish, inhibiting exploratory behavior by inducing myofiber loss, inhibiting muscle contraction, and causing changes in endurance and velocity. Thus, the zebrafish model can be used to screen pharmaceutical agents and to study muscle atrophy. Furthermore, maca is a potential dietary supplement to prevent muscle atrophy, as it protects muscle fibers.

## 1. Introduction

In the human body, skeletal muscle is essential for the homeostasis of metabolic parameters, such as basal metabolic rate, glucose uptake, and lipid utilization [1]. In addition, the balance of homeostasis between protein synthesis and degradation maintains muscle mass within myofibers [2]. Abnormal regulation of such fibers can interfere with the contractile properties of myofibers, rendering them less stable and more susceptible to contraction-induced damage, which eventually leads to muscle atrophy [3]. Therefore, the reduction in skeletal muscle mass and myofiber size is associated with several metabolic disorders, including pathological fractures and functional deterioration [4].

Muscle atrophy refers to the loss of skeletal muscle mass, strength, and myofiber size due to a range of physiological conditions, including denervation, immobilization, corticosteroid exposure, and aging [5]. Muscle inactivity refers to a reduction in muscle tension and contraction [6]. The dysfunction of contractile proteins in muscle atrophy leads to impairment of force-generating capacity, which causes a reduction in muscle fiber size [7]. The excessive loss of muscle mass and impairment of muscle contractile function in muscle atrophy can be indicators of negative prognosis and impaired functional efficacy [7]. Therefore, there has recently been great interest in therapeutic agents that can prevent muscle atrophy and improve muscle strength [8,9].

Glucocorticoid, an endocrine hormone secreted from the adrenal cortex, is an important mediator of muscle protein degradation and upregulation of the ubiquitin proteasome pathway in skeletal muscle [10]. High doses and prolonged use of dexamethasone, a synthetic glucocorticoid analogue, have been reported to cause muscle atrophy [11,12]. In addition, excess levels of glucocorticoids act as negative regulators of hippocampal neurogenesis and have been found to be closely related to the impairment of the ability of the glucocorticoid receptor in dexamethasone-induced muscle atrophy [13,14]. Several studies have emphasized the role of glucocorticoids in skeletal muscle, increasing hippocampus vulnerability, and it may also aggravate motor neurodegeneration [15,16].

The biological half-life of glucocorticoids, including dexamethasone, generally reflects the rate of degradation of the steroid and, hence, is probably related to the duration of activity and the metabolic stability at the receptor site [17]. Dexamethasone has an activity of 36–54 h, which allows daily or twice daily dosing, determined by the duration of suppression of hypothalamic–pituitary–adrenocortical secretory activity [17,18]. Their relatively long-term biological activity is expected to prolong anti-inflammatory and hypothalamic-pituitary-adrenal suppressibility [19]. However, excess dexamethasone activates the intracellular glucocorticoid receptor-mediated mechanism, followed by translocation to the nuclei to induce the pathogenesis of glucocorticoid-induced skeletal muscle atrophy [20]. Involvement of several pathways to induce protein degradation in skeletal muscle has been observed in glucocorticoid-induced muscle atrophy, including the activation of transcription factor FOXO and upregulation of the expression of muscle-specific ubiquitin ligases, such as atrogin-1 and MURF1 [10].

Consequently, dexamethasone has been used to understand the mechanism underlying muscle atrophy in rat, mouse, and in vitro models [2,16,21,22]; however, a zebrafish model of dexamethasone-induced muscle atrophy has not yet been reported. As the changes in the muscle functions of zebrafish elicit associated changes in exploratory behavior, endurance, and velocity, as well as in the metabolic and endocrine systems [23], zebrafish is suitable for modeling the histological and behavioral effects of muscle atrophy. Glucocorticoid receptor gene, which is responsible for muscle atrophy, is known to have a relatively high level of similarity with its human equivalent; therefore, zebrafish could be a valuable model for muscle atrophy research [24]. In addition, glucocorticoid receptor signaling is affected in adult fish but not in larvae, as the circuitry fully develops after the larval stages in zebrafish [25]. Furthermore, these species offer a potentially cheaper and simpler alternative to classical laboratory rodent species for modeling and understanding the mechanisms of human disorders, such as muscle atrophy, because they have external fertilization and a relatively short lifespan [26].

*Lepidium meyenii*, commonly referred to as maca or Peruvian ginseng, is used as a dietary supplement and has been shown to have anti-fatigue effects in different models of healthy mice by enhancing swimming endurance capacity [27,28]. Subsequently, we investigated the protective effect of maca on the decrease in myofiber size, the regulatory muscle protein troponin C and I levels, and the impaired exploratory behavior resulting from dexamethasone-induced muscle atrophy in zebrafish.

In this study, we focused on the physiological and behavioral aspects of zebrafish with dexamethasone-induced muscle atrophy and discussed methods that can be employed to quantify them. We then performed a physiological and behavioral study of muscle function in zebrafish with dexamethasone-induced muscle atrophy after pretreatment with maca. The data from this study suggest that the developed zebrafish model of muscle atrophy could be used for screening pharmaceutical agents and that maca is a potential candidate supplement for the prevention and treatment of muscle atrophy.

## 2. Materials and Methods

### 2.1. Materials

Dexamethasone, bovine serum albumin (BSA), eosin, ethyl 3-aminobenzoate methanesulfonate (MS-222), and DPX were purchased from Sigma-Aldrich (St. Louis, MO, USA). Hematoxylin was provided by DAKO (Carpinteria, CA, USA). Troponin I (ab47003) and troponin C (ab51106) were used following their secondary antibodies: goat anti-rabbit IgG H&L (Alexa Fluor^®^ 488, ab150077) or anti-rat IgG (H+L), Alexa Fluor^®^ 555 Conjugate (ab4417S), respectively, from Abcam (Cambridge, UK). The condensed maca (*Lepidium meyenii*), used in food supplements, was purchased from NOW Foods (Lot No. 3063322, Bloomingdale, IL, USA) as a 6:1 concentrate by starch removal from fresh maca.

### 2.2. Experimental Design and Animal Model

Animal care and experimental protocols were approved by the Animal Care and Use Committee of Jeju National University (approval no. 2019-0056). Adult, 1-year old zebrafish were purchased from a commercial dealer (Jeju Aquarium, Jeju, Korea). Wild-type zebrafish were maintained at a constant water temperature (28.5 ± 1 °C) with a 14 h:10 h light/dark cycle and were fed TetraBits complete twice per day (Tetra GmbH D-49304, Melle, Germany).

As zebrafish are capable of absorbing drug molecules through their skin and gills [29,30], dexamethasone solution was prepared by dissolving dexamethasone in water. To determine the concentration of dexamethasone solution, we performed a preliminary study in which we screened the toxicity of different concentrations of dexamethasone solution on zebrafish larvae, which is easily absorbed, and selected the range in which no significant toxicity was observed (data not shown). Briefly, the selected concentration was chosen because it significantly altered physiology but did not affect the survival or gross morphology. In addition, the dose-ranging study (0.0001–0.01%) was selected on the basis of a previously published study [31,32]. Dexamethasone has an activity of 36–54 h, which allows daily or twice daily dosing. We attempted to induce acute muscle atrophy in zebrafish by exposure to dexamethasone, but not at aggressive concentrations. This timing of exposure was selected on the basis of the optimal timing of exposure to dexamethasone in a previously published study [18].

Zebrafish (*n* = 84, *n* > 15 in each group) were randomly divided into four groups: non-treated group (control) as well as 0.0001%, 0.001%, and 0.01% dexamethasone-treated groups. After acclimation for 1 week, the zebrafish were placed in 0.0001%, 0.001%, and 0.01% dexamethasone solution for 1 h/day. After 3, 7, or 10 days, all zebrafish were sacrificed in 0.0006% MS-222 and the muscles were collected for hematoxylin and eosin (H&E) staining and immunofluorescence analysis.

In another set of experiments, zebrafish (*n* = 80, *n* > 15 in each group) were randomly divided into five groups: control, dexamethasone (0.01%)/saline, and dexamethasone (0.01%)/maca (0.3%, 1%, and 3%). After the zebrafish were pre-fed with maca supplements for 3 days, each group of zebrafish was placed in the dexamethasone solution for 1 h per day for 7 days and fed with or without maca supplements at the same time. After 10 days, all zebrafish were examined for their exploratory behavior, sacrificed in 0.0006% MS-222, and the muscles were collected for H&E and immunofluorescence staining.

### 2.3. Tissue Preparation

Muscle tissues from zebrafish were fixed in Bouin’s solution for 24 h. The fixed tissue was transferred into a graded alcohol series for dehydration and embedded in paraffin. The paraffin blocks were sliced to a thickness of 7 µm.

### 2.4. Hematoxylin and Eosin (H&E) Staining

Paraffin-embedded slides were deparaffinized in xylene for 90 min and successively incubated in EtOH and distilled water for rehydration. After rehydration, the slides were stained with hematoxylin for 90 s, washed three times with running water, and stained with eosin for 20 s. After three further washes, the slides were mounted using DPX mountant solution and analyzed under a microscope. To measure the myofiber diameter and CSA, all images were acquired from at least three independent muscles per experimental condition, and at least three randomly selected images per muscle were analyzed using Image J 1.46r software (Wayne Rasband, National Institutes of Health, Bethesda, MD, USA). They were evaluated for every myofiber contained entirely within the image.

### 2.5. Immunofluorescence (IF) Staining

Sectioned slides from each paraffin block were placed into the medium to dehydrate and cleared successively using xylene and an ethanol series (100%, 95%, 90%, 80%, and 70%). Subsequently, the slides were placed in 1× antigen retrieval solution, blocked with blocking buffer (serum in 5% BSA at an equivalent ratio) at 21 ± 1 °C (room temperature), and incubated with primary antibody (1:400) at 4 °C overnight. The slides were washed three times for 5 min with 1× PBS and incubated with Alexa Fluor conjugated secondary antibody (1:200) for 2 h at room temperature. After the slides were washed three times for 5 min with 1× PBS, the slides were mounted using DPX mountant solution and analyzed under a microscope.

### 2.6. Exploratory Behavior Tests

After 10 days, each group of zebrafish was transferred into a 1.5 L tank for 1 h to acclimate, and their swimming behavior was assessed from the side view using a video camera (Olympus Tough TG-5, Tokyo, Japan).

To analyze swimming behavior, zebrafish fed a normal diet were recorded for 5 min. The swimming pattern, active time (min), and distance moved (cm) were analyzed for each individual [33,34]. The distance moved was calculated frame by frame. To measure the velocity of responding to stimuli, the zebrafish were starved for half a day and their inclination to chase food when fed over a period of 8 s was recorded. Then, the velocity in the active time at which they were chasing food after the time the fish perceived the food was measured as the distance moved (cm) per second. Cases in which the fish failed to perceive feeding were excluded. All videos were analyzed using the video tracking software LoliTrack 4.2.1 (Loligo Systems, Viborg, Denmark).

### 2.7. Chemical Composition of Maca

The total polysaccharide content was determined using the phenol-sulfuric acid protocol described previously [35]. The monosaccharide composition was verified by high-performance anion exchange chromatography using a CarboPac^TM^ PA1 column (4.5 mm × 50 mm; Thermo Fisher Scientific, Waltham, MA, USA). The samples were acidified with 4 M trifluoroacetic acid (TFA). Subsequently, the monosaccharide composition was identified and verified by comparing its retention time with that of the standard mixture. The functional groups were determined using a Bruker FTIR, Alpha II (Bruker, Karlsruhe, Germany) instrument with a wavenumber range of 400–4000 cm^−1^. The molecular weight was determined using a DAWN Heleos II multi-angle light scattering and Optilab T-rEX refractive index detector (MALS-RI, Wyatt Technology, Santa Barbara, CA, USA) system equipped with a Shimadzu HPLC system connected to a PL Aquagel-OH MIXED-H (7.5 × 300 mm, Agilent Technologies, Santa Clara, CA, USA). The sample was filtered through a membrane filter of a pore diameter of 0.22 µm before being eluted with 0.5 mol/L NaCl at a flow rate of 0.5 mL/min. Data analysis was carried out using ASTRA 6 software (Wyatt Technology) with a refractive index increment (dn/dc) of 0.140 mL/g. The structure of the commercial-grade fucoidan was analyzed for comparison.

### 2.8. Statistical Analysis

All results were analyzed using GraphPad Prism 5 and evaluated using one-way analysis of variance and Dunnett’s multiple range tests. Differences were defined as NS (not significant), ^#^ *p* < 0.05, ^##^ *p* < 0.01, and ^###^ *p* < 0.001 compared with the control group, and * *p* < 0.05, ** *p* < 0.01, and *** *p* < 0.0001 compared with the dexamethasone/saline group.

## 3. Results and Discussion

### 3.1. Muscle Atrophy in Zebrafish Treated with Dexamethasone

A lack of physical activity due to injury or illness can contribute to tissue loss during muscle atrophy [36]. Moreover, inactivity-related changes in muscle atrophy involve the shrinkage of myofibers due to a net loss of proteins, which often leads to increased muscle loss during aging [37]. Therefore, maintaining healthy muscles plays a crucial role in the prevention of metabolic disorders and healthy aging.

However, glucocorticoids, including dexamethasone, have been widely used in studies to induce depression or anxiety in zebrafish through glucocorticoid receptor signaling [34,38]. Morikane et al. (2020) and Xin et al. (2020) reported the impact of glucocorticoids on debilitating psychiatric disorders through percutaneous absorption by exposing zebrafish to the test reagent solution [29,39]. Although no studies have investigated the use of glucocorticoids to induce muscle atrophy in the zebrafish model, due to the high level of similarity between human and zebrafish glucocorticoid receptors, this animal model can be a valuable tool in research aimed at elucidating the mechanisms underlying muscle atrophy [40].

In this study, as a muscle atrophy screening model, adult zebrafish were placed in a solution containing 0.0001%, 0.001%, and 0.01% dexamethasone for 1 h/day for 3, 7, or 10 days. We then investigated the changes in the frequency of different myofiber diameters and changes in the cross-sectional area (CSA) of myofibers in zebrafish compared with those in the control group. As shown in Supplementary Figure S1a–c, the myofiber diameter and myofiber CSA following exposure to dexamethasone for 3 days were plotted on a graph to ascertain the changes in myofibers of zebrafish. In the control group, myofibers with a diameter of 50 µm accounted for the highest frequency (26%) of all myofibers. Following treatment with dexamethasone for 3 days, there was a change in myofiber distribution to a lower diameter, depending on the dexamethasone concentration. After treatment with 0.01% dexamethasone, myofibers with a diameter of 40 µm accounted for the highest frequency (27%). In addition, the mean CSA of myofibers in the 0.001% and 0.01% dexamethasone groups was 1854.53 µm^2^ and 1623.96 µm^2^, respectively, compared with that in the control group (2349.31 µm^2^), indicating that the mean CSA reduction rate was significantly increased by 21.06% and 30.88%, respectively (**** *p* < 0.0001, Supplementary Figure S1c). Skeletal muscle atrophy is characterized by the decrease in the muscle fiber size. These findings correspond to the previous studies in rodent models, which reported a reduction in the myofiber mass and muscle strength/endurance in dexamethasone-induced muscle atrophy by protein degradation caused by glucocorticoids [8,21,22]. In addition, a human study suggested a significant association between corticosteroid usage and skeletal muscle weakness in adults with cystic fibrosis or chronic obstructive pulmonary disease [41,42].

**Figure 1:**
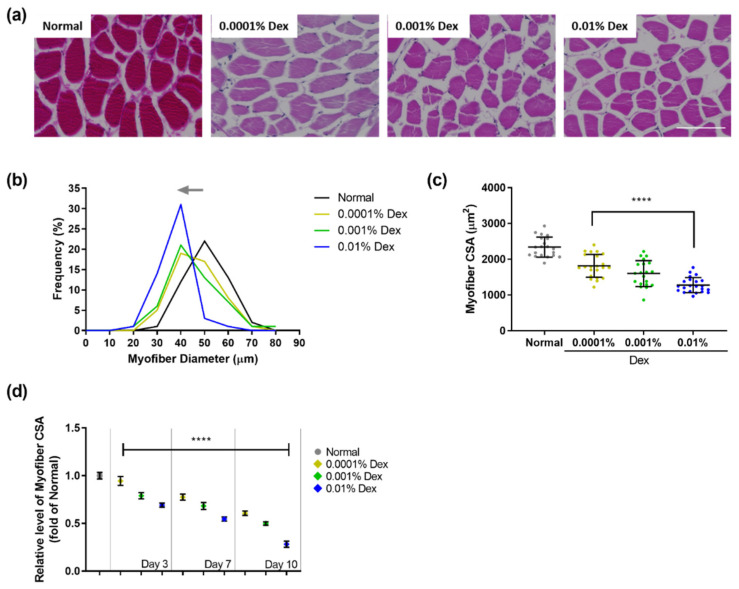
Validation by histological analysis of skeletal muscle tissue in the zebrafish model of dexamethasone-induced muscle atrophy. (**a**) Representative images using hematoxylin and eosin staining from muscle tissue of zebrafish exposed to dexamethasone (0.0001, 0.001, and 0.01%) for 7 days (scale bar = 100 µm). (**b**) Frequency of myofiber diameter and (**c**) myofiber CSA were quantified from representative images and analyzed using ImageJ software. (**d**) The relative levels of myofiber CSA with different dexamethasone concentrations (0.0001, 0.001, and 0.01%) for 3, 7, and 10 days normalized to the control group for each respective day. The data are presented as the mean ± SD. **** *p* < 0.0001 vs. control group.

After treatment with dexamethasone for 7 days, histological changes in the myofibers of zebrafish were evident (Figure 1a). The myofiber diameter in the control group, which was 50 µm for the highest frequency (22%), decreased significantly depending on the dexamethasone concentration (Figure 1b). The group treated with 0.01%, 0.001%, and 0.0001% dexamethasone contained 31%, 21%, and 19% of myofibers with a diameter of 40 µm, respectively. Moreover, in zebrafish exposed to 0.0001%, 0.001%, and 0.01% dexamethasone, the average CSA of myofibers was markedly reduced by 1814.00, 1600.26, and 1276.87 µm^2^ compared to that in the control group (2340.74 µm^2^) (**** *p* < 0.0001, Figure 1c).

Exposure to dexamethasone for 10 days induced critical changes in the myofibers of zebrafish (Supplementary Figure S2a). In particular, zebrafish treated with 0.01% dexamethasone for 10 days showed excessive changes in the diameter of muscle myofiber/myofiber CSA. Excessive histological changes in muscle impair myofiber homeostasis and functional efficacy and are indicators of prognosis [43]. We observed a remarkable shift toward smaller myofiber diameter and reduced CSA in the dexamethasone-treated groups compared to the control group (Supplementary Figure S2b,c).

The relative myofiber CSA after treatment with dexamethasone (0.0001%, 0.001%, and 0.01%) for 3, 7, and 10 days are shown in Figure 1d. As the dose of dexamethasone and the length of treatment increased, myofiber CSA tended to decrease. Therefore, the dosage regimen that showed a moderate effect, 0.01% dexamethasone for 1 h/day for 7 days, was selected to assess the therapeutic efficacy of maca. These results demonstrated that dexamethasone induced changes in morphological features of zebrafish, such as myofiber diameter and CSA, within a relatively short period of time.

### 3.2. Characterization of the Polysaccharides Derived from Maca

In the present study, we found that the dry weight of maca comprised a significant 40.10% ± 3.19% proportion of non-starch polysaccharide.

**Figure 2:**
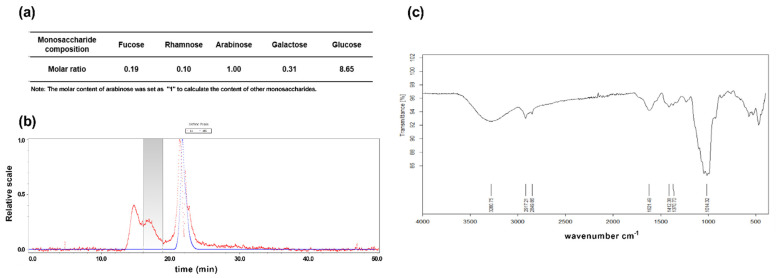
Physical and chemical characterization of maca. (**a**) High performance anion exchange chromatogram and monosaccharide composition of maca. The molar content of arabinose was set as “1” to calculate the content of other monosaccharides. (**b**) IR and (**c**) MALS spectrum of maca.

To determine the physical and chemical properties of maca, we performed high-performance anion exchange chromatography to analyze the monosaccharide composition of maca, which was found to be composed of fucose, rhamnose, arabinose, galactose, and glucose in molar ratios of 0.19, 0.10, 1.00, 0.31, and 8.65, respectively (Figure 2a). In addition, we performed FT-IR analysis to characterize the functional bonds and MALS analysis to determine the average molecular weight (Figure 2b).

The FT-IR spectrum of maca showed the typical signals of polysaccharides in the 4000–400 cm^−1^ range (Figure 2b). The broad band O-H stretching vibration indicated the presence of hydrogen bonds at 3445 cm^−1^ [44], whereas the band at 2928 cm^−1^ could be ascribed to the stretching vibration of C-H [45]. Furthermore, the peak at 1618 cm^−1^ can be attributed to C=O stretching [46] and the strong band in the 1200–800 cm^−1^ wavenumber region is assumed to be associated with monosaccharides such as arabinose, fructose, galactose, glucose, and mannose [47]. According to the MALS-RI analysis in Figure 2c, the weight-average molecular weight (M_w_), number-average molecular weight (M_n_), and z-average molecular weight (M_z_) values of maca were found to be 9.836 × 10^4^, 1.022 × 10^5^, and 1.066 × 10^6^ g/mol, respectively. Furthermore, the maca had a narrow mass distribution, as evidenced by the M_w_/M_n_ value of 1.039 [48].

The biological activity of polysaccharides is largely determined by the monosaccharide composition and molecular weight [46]. In this regard, the findings of previous studies indicated that the anti-fatigue activity attributed to polysaccharides is closely associated with glucose content [49]. Accordingly, we speculate that the high glucose content of maca may have contributed to the alleviation of muscle atrophy dysfunction observed in the present study. In addition, Liu et al. (2010) previously detected a correlation between the low molecular weight of polysaccharides and their biological activity [50]. Given that the molecular weight of polysaccharides can typically reach values in the range of hundreds of kDa, the polysaccharide of 9.836 × 10^4^ Da obtained in the present study can be considered a relatively low-molecular-weight polysaccharide. In contrast to high molecular weight polysaccharides, orally administered polysaccharides of low molecular weight can be absorbed across the intestinal lining into the bloodstream and have systemic biological effects [51].

### 3.3. Effect of Maca in Myofibers of Zebrafish with Muscle Atrophy

Maca is known to exert pharmacological effects, notably by improving endurance capacity and motor coordination through its anti-fatigue effects [28,52]. To date, however, studies have been limited to examining the effects of maca on biological parameters related to the anti-fatigue state in different types of healthy rodent models [28,46,49]. In the present study, we investigated the effects of a low molecular weight polysaccharide of maca comprising a high proportion of glucose on the myofibers of muscles, their associated proteins, and the behavioral changes in zebrafish with dexamethasone-induced muscle atrophy.

**Figure 3:**
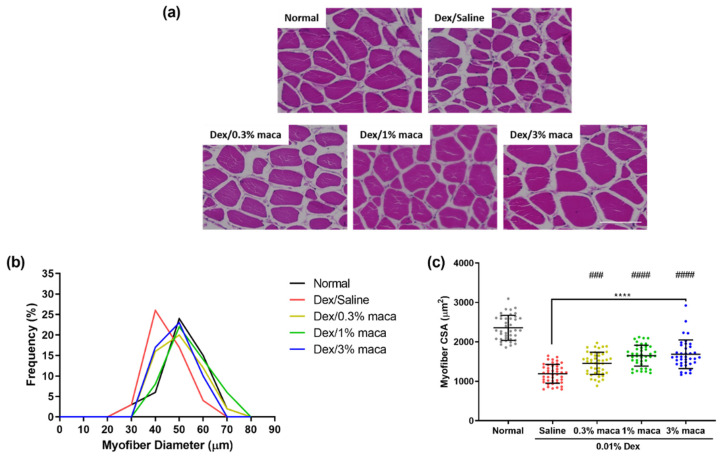
The effect of maca (0%, 1%, and 3%) on the histology of skeletal muscle tissue in zebrafish exposed to 0.01% dexamethasone for 7 days. (**a**) Representative images of hematoxylin and eosin staining showing histological changes in the muscle tissue of zebrafish (scale bar = 100 µm). (**b**) Frequency of different diameters of myofibers and (**c**) myofiber CSA was quantified from representative images and analyzed using ImageJ software. The data are presented as the mean ± SD. **** *p* < 0.0001 vs. control group. ^###^ *p* < 0.001 and ^####^ *p* < 0.0001 vs. dexamethasone/saline group.

Based on the model established in this study, muscle atrophy was induced by immersion of the zebrafish in a solution of 0.01% dexamethasone for 7 days, and the effect of maca pretreatment (0.3%, 1%, and 3%) for 3 days was investigated by H&E staining (Figure 3a). Maca pretreatment protected the myofibers of zebrafish from dexamethasone-induced muscle atrophy compared to that in the group not pretreated with maca (dexamethasone/saline). In the dexamethasone/saline group, the diameter was reduced to 40 µm at a 26% frequency of myofibers (Figure 3b). However, in all the maca-treated groups (20%, 22%, and 23%, respectively), a myofiber diameter of 50 µm was observed, which was similar to that in the control group (24%), which showed the remarkable protective ability of maca against dexamethasone-induced muscle atrophy. As shown in Figure 3c, the CSA of myofibers in the dexamethasone/saline group, which was reduced by approximately 49.47% (1190.92 µm^2^, **** *p* < 0.0001) compared to that in the control group, also considerably improved to 38.22%, 29.99%, and 28.44% at 1456.02, 1650.01, and 1686.55, respectively, at 0.3%, 1%, and 3% maca pretreatment. Additionally, pretreatment with maca at 1% and 3% significantly increased myofiber CSA, respectively, compared to that in the dexamethasone/saline group (^####^ *p* < 0.0001). These results showed that maca pretreatment exerted a protective effect against dexamethasone-induced muscle atrophy in zebrafish, protecting myofibers by preventing the reduction in myofiber size.

### 3.4. Effect of Maca on Muscle Contractile Proteins in Zebrafish with Muscle Atrophy

Skeletal muscle filaments consist of various components, including actin, tropomyosin, and troponin (Tn) complex, which regulate the activation of calcium-dependent myofilaments and force-producing myosin–actin interactions, and control the myofilament protein interactions that mediate muscle contraction/relaxation [53,54]. The Tn complex in myofibers is composed of a calcium-binding subunit, troponin C (TnC); an actin-binding inhibitory subunit, troponin I (TnI); a tropomyosin (Tm)-binding subunit; and troponin T (TnT). TnC contains a Ca^2+^-binding site, which leads to increased calcium sensitivity and changes in the duration of contraction by enhancing calcium–TnC affinity, thereby reducing the calcium dissociation rate, which in turn stimulates the affinity of TnI for TnC, allowing actin to interact with myosin, resulting in muscle contraction [55]. In contrast, TnI binds to actin in the relaxed state, preventing muscle contraction by inhibiting the ATPase activity of actomyosin [56].

**Figure 4:**
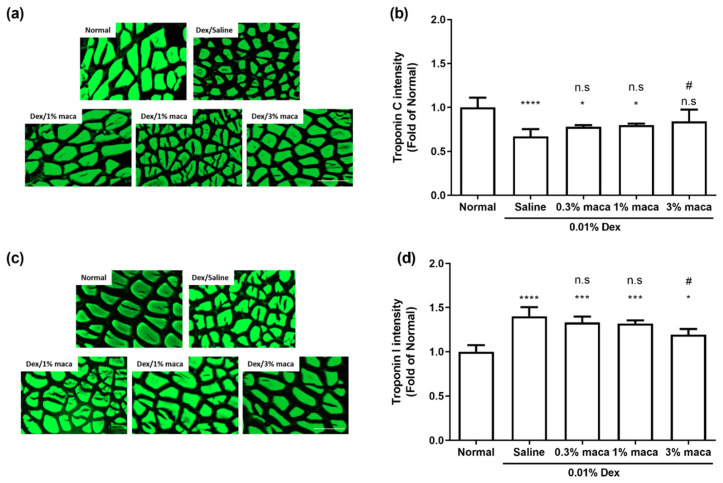
The effect of maca (0.3%, 1%, and 3%) on contractile proteins of skeletal muscle tissue in zebrafish exposed to 0.01% dexamethasone for 7 days. (**a**) Representative immunofluorescence images show the TnC expression in the muscle tissue of zebrafish (scale bar = 100 µm). (**b**) The graph quantifies the relative intensity of TnC from representative images and was analyzed using ImageJ software. (**c**) Representative immunofluorescence images show TnI expression in the muscle tissue of zebrafish (scale bar = 100 µm). (**d**) The graph quantifies the relative intensity of TnI from representative images and was analyzed using ImageJ software. The data are presented as the mean ± SD. * *p* < 0.05, *** *p* < 0.001, and **** *p* < 0.0001 vs. control group. ^#^ *p* < 0.05 vs. dexamethasone/saline group.

Thus, we investigated the dysfunction of the contractile proteins TnC and TnI in zebrafish with dexamethasone-induced muscle atrophy and examined the effect of maca on contractile proteins. As shown in Figure 4a,d, the mutually exclusive trend of TnC and TnI in the control group was found to be reversed in the dexamethasone/saline group (**** *p* < 0.0001). This shows that dexamethasone can impair the Tn complex and interfere with calcium binding to the Tn complex, which is believed to regulate muscle contraction. The correlation between increased TnI and skeletal muscle weakness due to drug-induced myopathy has been found in in vivo or clinical studies [57,58,59]. Besides, in the muscles of hindlimb unloading rats, TnC showed decreased muscle atrophy due to muscle-type specificity [60]. However, the impairment of TnC and TnI by dexamethasone was prevented in the dexamethasone/maca groups. In particular, 3% maca pretreatment significantly inhibited TnC and TnI compared to that in the dexamethasone/saline group (^#^ *p* < 0.1, TnC and TnI), which improved to a level similar to that of the control group (n.s. and * *p* < 0.1, TnC and TnI, respectively). These results suggest that maca prevents the impairment of the muscle contractile proteins TnC and TnI, which might protect against damage induced by muscle atrophy.

### 3.5. Effect of Maca on Exploratory Behavior in Zebrafish with Muscle Atrophy

Zebrafish have been used for the study of muscle cell biology, function, and a range of muscle diseases, including dystrophies and congenital myopathies. A reduction in the movement of zebrafish, notably in activity, distance, and velocity, indicates skeletal muscle dysfunction [61]. The understanding of behavioral phenotypes of zebrafish in a fish tank provides insights into physiological responses related to their sensitivity to pharmacological and genetic factors or pathological function [62]. Active swimming, exploring throughout the tank, chasing/ingesting food is typically interpreted as reflecting fish that feel comfortable and relaxed [62]. In addition, food seeking/chasing is a common foraging behavior of zebrafish which is triggered by hunger [62]. Following the ethogram of zebrafish behavior, we observed the behavioral response of the zebrafish exposed to dexamethasone or the changes after pretreatment with maca. Furthermore, we examined whether there was a mechanistic link between the impairment of muscle contractile proteins and exploratory behavior in the zebrafish with dexamethasone-induced muscle atrophy, which altered muscle contractile properties to cause changes in sustained exploratory behavior, by examining voluntary movements over a limited time and chasing velocity [63]. One limitation of this technique is that the determination of sustained interest in swimming performance is subjectively assessed by the observer. However, the swimming performance of zebrafish was assessed over a limited time period with repeated or randomized selection in our study.

**Figure 5:**
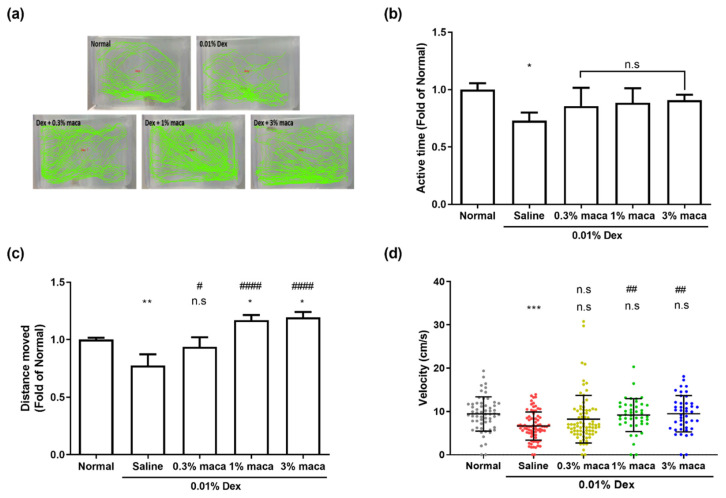
The effect of maca (0.3%, 1%, and 3%) on the swimming behavior of zebrafish exposed to 0.01% dexamethasone for 7 days. The swimming behavior of zebrafish was tracked at the end of each analysis using LoliTrack software. (**a**) Swimming patterns recorded for 10 min (shown in green highlight) were measured for the following: (**b**) and the active time (**c**) the average distance moved (**d**). The velocity of each zebrafish was measured in cm per second in the active state for food tracking. The data are presented as the mean ± standard deviation. * *p* < 0.05, ** *p* < 0.01 and *** *p* < 0.001 vs. control group. ^#^ *p* < 0.05, ^##^ *p* < 0.01 and ^####^ *p* < 0.0001 vs. dexamethasone/saline group.

As shown in Figure 5, compared with the control group, the dexamethasone/saline group with altered muscle contractile proteins exhibited changes in endurance, physical activity, and swimming performance, indicating that zebrafish with dexamethasone-induced muscle atrophy showed decreased exploratory behavior (Figure 5a,b, * *p* < 0.1). In the control group, the zebrafish swam all over the tank, with increased active time and longer distance moved, whereas zebrafish in the dexamethasone/saline group tended to swim at the bottom of the tank with less activity. In the maca group, there was no significant change in the active time, despite a slight increase compared with the dexamethasone/saline group; however, an increase in the distance moved was observed with a swim pattern throughout the entire tank (Figure 5c). After dexamethasone treatment, the distance moved significantly decreased to 0.77, compared to that in the control group (** *p* < 0.01), but the 1% and 3% maca groups significantly traveled to distances of 1.17 and 1.19, respectively, which was higher than that in the control group (* *p* < 0.1), suggesting that maca pretreatment could improve muscle endurance and prevent the reduction of movements observed in the dexamethasone/saline group.

As shown in Figure 5c, to measure the velocity in response to stimuli, when analyzing the velocity at which the zebrafish chased food, we found that dexamethasone treatment resulted in a significant reduction in velocity compared with that in the control group (*** *p* < 001), indicating that dexamethasone reduces the reaction speed and muscle strength to stimulation. However, 1% and 3% maca pretreatment significantly prevented a reduction in velocity compared with that observed in the dexamethasone/saline group (^##^ *p* < 0.01). The change in velocity for each group is shown in Supplementary Video S1. These results indicate that maca has a protective effect on swimming endurance and muscle strength in zebrafish with muscle atrophy induced by dexamethasone.

## 4. Conclusions

In conclusion, in this study, zebrafish exposed to dexamethasone, which is known to induce muscle atrophy, was suggested as a muscle atrophy model. Changes in muscle fiber loss, damage to muscle contractile proteins, and abnormal exploratory behavior due to structural and functional impairment of the skeletal muscle associated with muscle atrophy were investigated. Additionally, using this zebrafish model of muscle atrophy, we investigated the effect of maca on myofiber loss and muscle contractile proteins that determine muscle function. We report the suitability of the dexamethasone-induced zebrafish model for the investigation of glucocorticoid-induced skeletal muscle atrophy and observed that maca pretreatment can prevent or reverse the changes caused by muscle atrophy. We believe that these findings can contribute to the screening and discovery of materials that prevent skeletal muscle homeostasis, recovery, or muscle atrophy.

## Data Availability

The datasets generated and/or analyzed during the current study are available from the corresponding author upon reasonable request.

## Supplementary Materials

The following are available online at https://www.mdpi.com/article/10.3390/cells10112879/s1, Figure S1: Histological analysis of skeletal muscle tissue in a zebrafish model exposed to dexamethasone for 3 days. Figure S2: Histological analysis of skeletal muscle tissue in a zebrafish model exposed to dexamethasone for 10 days. Control: non-treated zebrafish; saline, 0.01% Dex; zebrafish exposed to 0.01% dexamethasone for 7 days; 0.3% maca, 1% maca, and 3% maca with 0.01% Dex; zebrafish pre-fed with maca (0.3%, 1%, and 3%) for 3 days exposed to 0.01% dexamethasone for 7 days. Video S1: Recorded velocity of zebrafish exposed to 0.01% dexamethasone for 7 days and with maca pretreatment (0.3%, 1%, and 3%).
