# Supplementary material for: Zebrafish Model for Studying Dexamethasone-Induced Muscle Atrophy and Preventive Effect of Maca (Lepidium meyenii)
Source: Cells. 2021 Oct 25;10(11):2879. doi: 10.3390/cells10112879 (PMC8616435; doi:10.3390/cells10112879)
Supplement: Supplementary file 1 [file cells-10-02879-s001.zip › supplementary data/videos.pptx]

## Slide 1
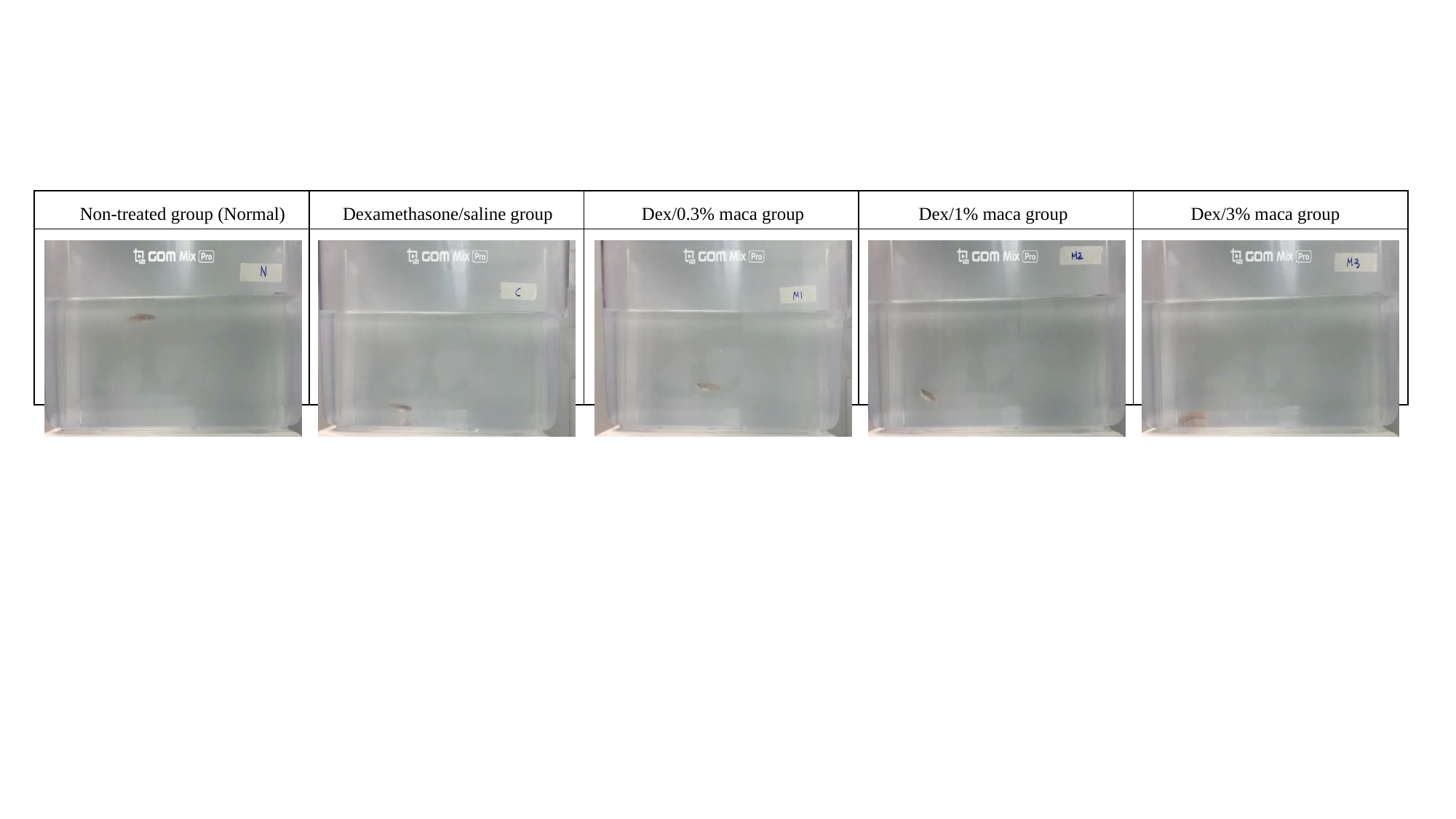

| | | | | |
| --- | --- | --- | --- | --- |
| | | | | |
Dex/1% maca group
Dex/3% maca group
Dexamethasone/saline group
Dex/0.3% maca group
Non-treated group (Normal)
