# Supplementary figures and images for: Zebrafish Model for Studying Dexamethasone-Induced Muscle Atrophy and Preventive Effect of Maca (Lepidium meyenii)
Source: Cells. 2021 Oct 25;10(11):2879. doi: 10.3390/cells10112879 (PMC8616435; doi:10.3390/cells10112879)

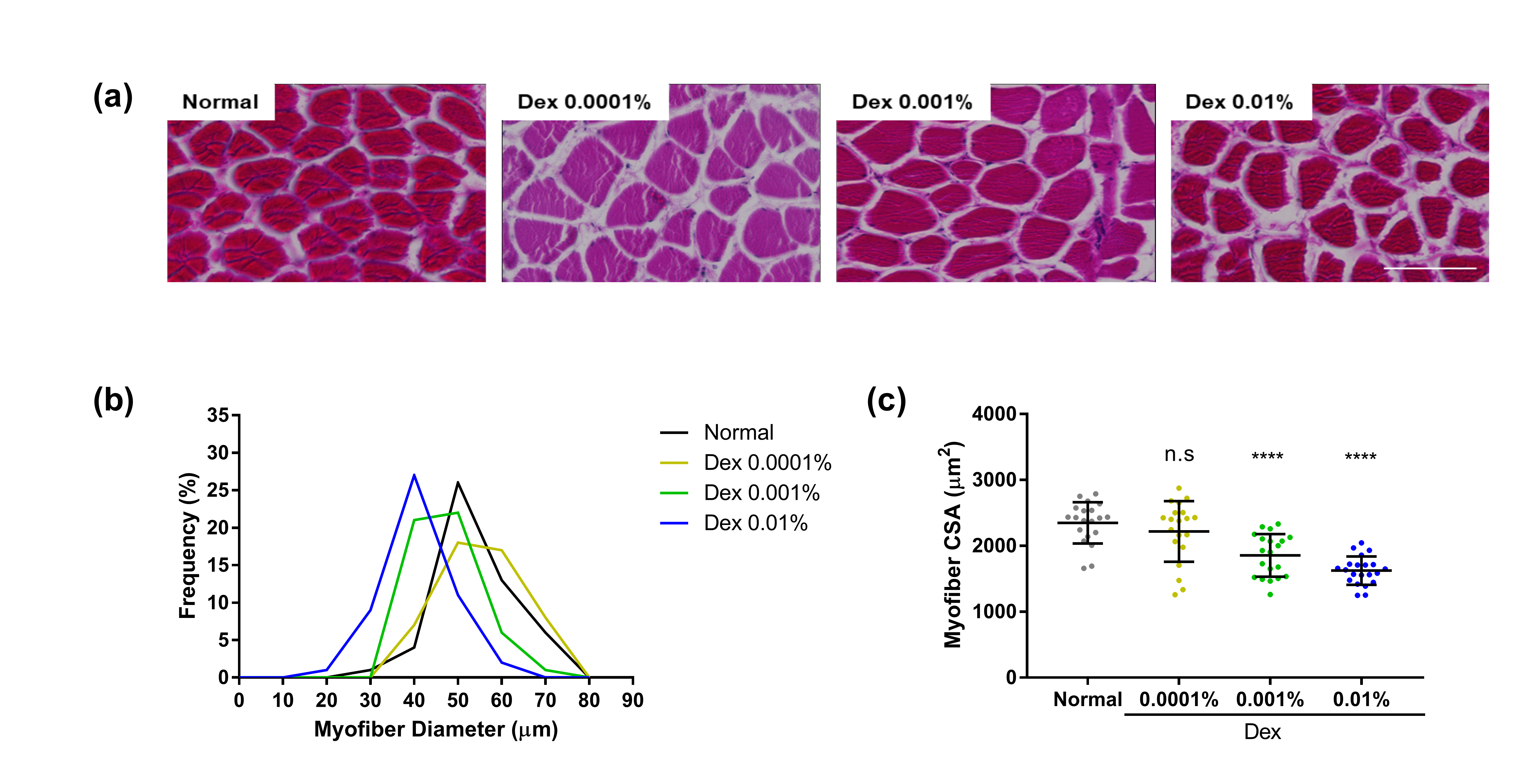

Supplement: Supplementary file 1 [file cells-10-02879-s001.zip › supplementary data/S1.png]

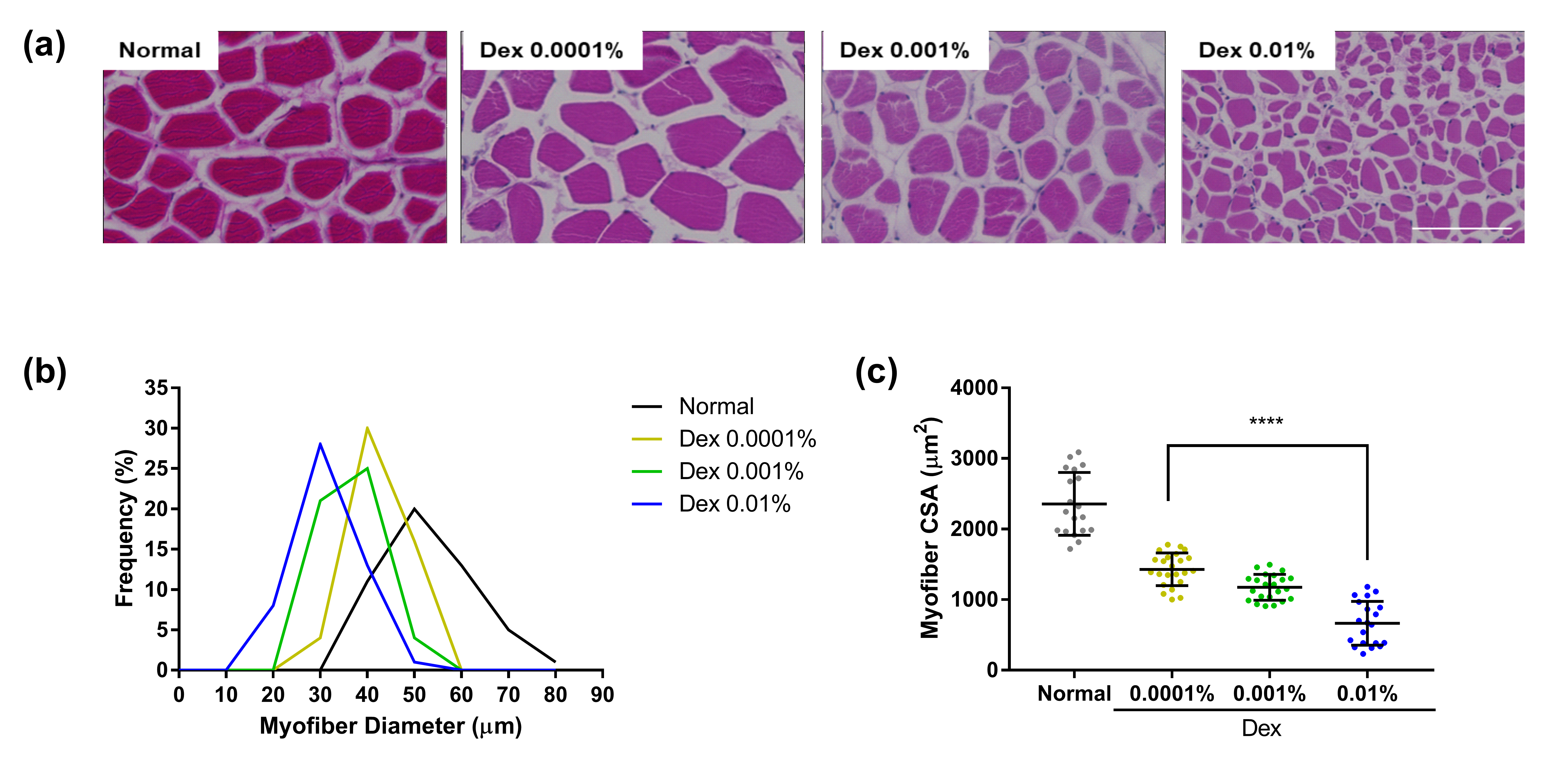

Supplement: Supplementary file 1 [file cells-10-02879-s001.zip › supplementary data/S2.png]
